# Influence of CO_2_ Degassing on the Microbial Community in a Dry Mofette Field in Hartoušov, Czech Republic (Western Eger Rift)

**DOI:** 10.3389/fmicb.2018.02787

**Published:** 2018-11-21

**Authors:** Qi Liu, Horst Kämpf, Robert Bussert, Patryk Krauze, Fabian Horn, Tobias Nickschick, Birgit Plessen, Dirk Wagner, Mashal Alawi

**Affiliations:** ^1^GFZ German Research Centre for Geosciences, Section Geomicrobiology, Potsdam, Germany; ^2^GFZ German Research Centre for Geosciences, Section Organic Geochemistry, Potsdam, Germany; ^3^Institute of Applied Geosciences, Technische Universität Berlin, Berlin, Germany; ^4^Institute for Geophysics and Geology, University of Leipzig, Leipzig, Germany; ^5^GFZ German Research Centre for Geosciences, Section Climate Dynamics and Landscape Evolution, Potsdam, Germany; ^6^Institute of Earth and Environmental Science, University of Potsdam, Potsdam, Germany

**Keywords:** geo–bio interaction, elevated CO_2_ concentration, paleo-sediment, deep biosphere, acidophilic microorganisms, *Acidobacteriaceae*, *Acidithiobacillus*, *Acidothermus*

## Abstract

The Cheb Basin (CZ) is a shallow Neogene intracontinental basin filled with fluvial and lacustrine sediments that is located in the western part of the Eger Rift. The basin is situated in a seismically active area and is characterized by diffuse degassing of mantle-derived CO_2_ in mofette fields. The Hartoušov mofette field shows a daily CO_2_ flux of 23–97 tons of CO_2_ released over an area of 0.35 km^2^ and a soil gas concentration of up to 100% CO_2_. The present study aims to explore the geo–bio interactions provoked by the influence of elevated CO_2_ concentrations on the geochemistry and microbial community of soils and sediments. To sample the strata, two 3-m cores were recovered. One core stems from the center of the degassing structure, whereas the other core was taken 8 m from the ENE and served as an undisturbed reference site. The sites were compared regarding their geochemical features, microbial abundances, and microbial community structures. The mofette site is characterized by a low pH and high TOC/sulfate contents. Striking differences in the microbial community highlight the substantial impact of elevated CO_2_ concentrations and their associated side effects on microbial processes. The abundance of microbes did not show a typical decrease with depth, indicating that the uprising CO_2_-rich fluid provides sufficient substrate for chemolithoautotrophic anaerobic microorganisms. Illumina MiSeq sequencing of the 16S rRNA genes and multivariate statistics reveals that the pH strongly influences microbial composition and explains around 38.7% of the variance at the mofette site and 22.4% of the variance between the mofette site and the undisturbed reference site. Accordingly, acidophilic microorganisms (e.g., OTUs assigned to *Acidobacteriaceae* and *Acidithiobacillus*) displayed a much higher relative abundance at the mofette site than at the reference site. The microbial community at the mofette site is characterized by a high relative abundance of methanogens and taxa involved in sulfur cycling. The present study provides intriguing insights into microbial life and geo–bio interactions in an active seismic region dominated by emanating mantle-derived CO_2_-rich fluids, and thereby builds the basis for further studies, e.g., focusing on the functional repertoire of the communities. However, it remains open if the observed patterns can be generalized for different time-points or sites.

## Introduction

Due to magmatic activity beneath the Cheb Basin, large-scale degassing of mantle-derived CO_2_ (>99%) occurs. The diffuse cold gas emanations at the surface (diffuse degassing structures, DDS) can be distinguished as dry and wet mofettes (Kämpf et al., 2013). Mofettes provide insights into life under elevated CO_2_ concentrations, low pH, and anoxic conditions comparable to the Earth’s ancient atmosphere (Emiliani, 1992; Raven, 1995; Young et al., 2012). Moreover, mofettes are used as model ecosystems for studying the response of soil microorganisms to a potential CO_2_ leakage of underground carbon capture and storage systems (Krüger et al., 2009, 2011; Frerichs et al., 2013; Morales and Holben, 2013).

CO_2_ degassing leads to hypoxia and acidification of the soil (Beaubien et al., 2008; Blume and Felix-Henningsen, 2009; Rennert and Pfanz, 2016). Additionally, an increase in metal mobilization was observed, which may affect the availability of soil nutrients (Mehlhorn et al., 2014, 2016). As shown by several studies, these direct influences of elevated CO_2_ concentrations on the environment are affecting the mofette biota and its biological matter cycling.

First studies on the effects of CO_2_ on the soil biota were focused on the plant vegetation. These studies point to a decelerated growth, an increased plant C/N ratio but also physiological adaptations that provide advantages in hypoxic or even anoxic environments (Pfanz et al., 2004; Vodnik et al., 2007; Rennert and Pfanz, 2016). A common feature of mofette soils are increased carbon and nitrogen contents (Ross et al., 2000; Rennert et al., 2011). By δ^13^C analyses of plant and microbial lipids, Oppermann et al. (2010) demonstrated that within a CO_2_ vent in the Latera Caldera (Central Italy) a substantial amount of geothermal CO_2_ is incorporated into the microbial, plant, and soil carbon pools. Recently, Beulig et al. (2016) performed radiocarbon analyses and showed that up to 67% of mofette soil carbon content originated from the assimilation of geogenic CO_2_ via plant primary production and microbial CO_2_ fixation. The authors pointed out that the almost undegraded organic material found in the mofette soil is facilitated by the permanent exclusion of meso- to macroscopic eukaryotes rather than an impaired biochemical potential for soil organic matter decomposition. Complementary, Nowak et al. (2015) estimated through combined δ^14^C and δ^13^C isotope mass balances that around 8–27% of the bulk soil organic matter (SOM) derived from microorganisms. DNA stable isotope probing allowed the identification of chemolithoautotrophic microorganisms such as methanogenic archaea and acetogens as well as sulfate reducing bacteria (SRB) to be involved in the assimilation of CO_2_ (Oppermann et al., 2010; Beulig et al., 2015). However, a quantification of *cbbL* genes, encoding for the large subunit of RuBisCO, a carboxylase which is of crucial importance for carbon assimilation in chemolithoautotrophic microbes, revealed that only a part of the autotrophic CO_2_-fixing microorganisms could adapt to the very high CO_2_ concentrations found in a mofette in Slovenia (Videmšek et al., 2009).

In the last decade, several studies have focused on the impact of elevated CO_2_ concentrations on the microbial community structure in mofettes. A shift to anaerobic/microaerophilic and acidophilic community compositions has been reported in CO_2_ mofette soils located near the Laacher See (Germany) (Krüger et al., 2009; Krüger et al., 2011), the Cheb Basin (Czech Republic) (Beulig et al., 2015), and in Stavešinci (Slovenia) (Videmšek et al., 2009). Furthermore, at the Laacher See site, the abundance of several functional and group-specific gene markers revealed a decrease in *Geobacteraceae* and an increase in SRBs in the vent center and biomarker analysis revealed a predominance of *Thaumarchaeota* as possible indicator organisms for elevated CO_2_ concentrations in soils (Frerichs et al., 2013). Also for a mofette in Latera, Italy, it was shown that strictly anaerobic SRBs are abundant in mofettes, whereby the ATP biomass and total bacterial cell counts decreased (Beaubien et al., 2008). The highest sulfate reducing activity was observed in the center of the vent. Also Methanogenic archaea showed higher activities in the center of the vent compared to a transition zone site. Contrary to the results from Frerichs et al. (2013) in the Laacher See mofette, *Geobacteraceae* were in Latera mainly found at the CO_2_ vent and only minor quantities were found at the reference site (Oppermann et al., 2010).

So far, only a few population datasets from dry mofettes are based on high throughput sequencing. The 454 pyrosequencing analyses of 16S rRNA genes from a natural mofette in central-southern Spain revealed that community richness, evenness, and diversity decreased with increasing CO_2_ flux (Sáenz de Miera et al., 2014). An increase in abundance was thereby observed for OTUs related to the *Chloroflexi* phylum. Interestingly, Beulig et al. (2015) also showed that besides *Methanoregulaceae*, unclassified *Chloroflexi* might be involved in acetogenesis by DNA-SIP. The second 16S rRNA pyrosequencing dataset was established by Beulig et al. (2015) for a mofette located in the Plesná floodplain in the Cheb Basin. The community was dominated by methanogens (e.g., *Methanosarcinales* and *Methanomicrobiales*) and subdivision 1 *Acidobacteria*, which likely thrived under stable hypoxia and acidic pH. Recently, Krauze et al. (2017) investigated wet mofettes in the Cheb Basin by Illumina 16S rRNA amplicon sequencing and found a unique microbial community highly adapted to the anoxic conditions in the mineral and thermal waters and highlighted the connection between the groundwater or mineral waters and the deep biosphere. Deeper insights into the influence of CO_2_ on the microbial community within the top 40 cm of a mofette in the Cheb Basin were gained by a metatranscriptomic and metagenomic approach (Beulig et al., 2016). One outcome of the study was that transcripts related to methanogenesis (*mcr*) and sulfate reduction (*dsr*, *cys*, *apr*) were remarkably increased in frequency.

However, until now, microbiological studies have focused only on near-surface soil layers (<70 cm) of dry mofettes, and deeper sediments have not yet been analyzed in detail. The present study aims to characterize the influence of elevated CO_2_ concentrations inside a CO_2_ conduit on the geochemistry, microbial abundance, and community composition. Furthermore, this study intends to determine significant community-shaping environmental factors. To study the influence of mantle-derived CO_2_, two 3-m drillings were performed, one of which was located in the center of the DDS and the other at the undisturbed border of the mofette field. To unravel the community structures a high resolution sampling (every 5 to 10 cm) and Illumina 16S rRNA gene amplicon sequencing was conducted.

## Materials and Methods

### Site Selection, Description, and Sampling

**FIGURE 1:**
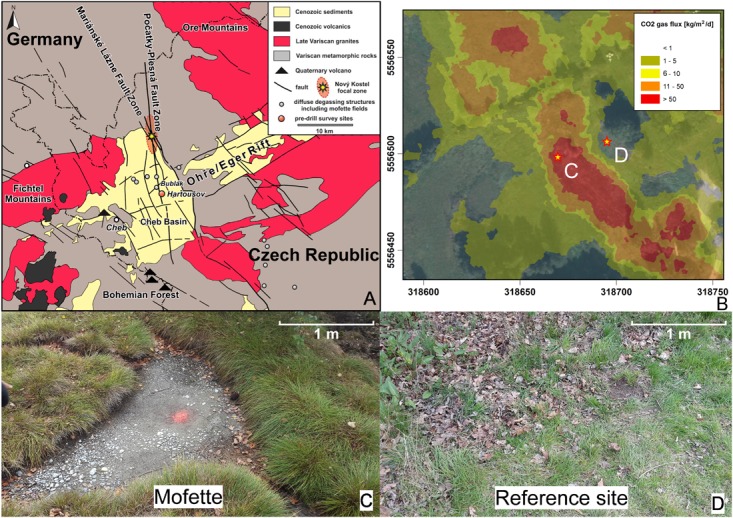
Geological map of the Cheb Basin (modified from Flechsig et al., 2008) **(A)**, CO_2_ gas flux in the Hartoušov mofette field (modified from Nickschick et al., 2015, coordinates in UTM 33N) **(B)**, surface at the mofette **(C)** and the reference site **(D)**. The drilling locations are marked by stars (**C** = mofette site; **D** = reference site) in the CO_2_ gas flux map **(B)**.

The drilling campaign was conducted in September 2015 in the Hartoušov Mofette Field (HMF; 50°07′58′′N, 12°27′46′′E; Figure 1B). The study site is located in the Cheb Basin (NW Bohemia, Czechia), a shallow Neogene intracontinental basin filled with fluvial and lacustrine sediments (≤350 m thick; Figure 1A). The basin formed at the intersection of the Eger Rift (Kopecký, 1979) and the Regensburg–Leipzig–Rostock fault zone (Bankwitz et al., 2003b; Geissler et al., 2004) where four Quaternary volcanoes existed (Mrlina et al., 2009; Rohrmüller et al., 2018). The western Eger Rift has been well studied regarding structure of the lithosphere, seismic activity, sedimentology, and fault characteristics (Dobeš et al., 1986; Bucha, et al, 1990; Špičáková et al., 2000; Bankwitz et al., 2003a; Kämpf et al., 2005, 2007, 2013; Flechsig et al., 2008; Fischer et al., 2014; Nickschick et al., 2015; Bussert et al., 2017). The seismic activity in this area occurs as “earthquake swarms,” which are typically numerous small earthquakes at upper crustal depths that cluster in time and space (Fischer et al., 2014). Earthquake swarms usually occur in volcanic areas, geothermal fields, and ocean ridges, whereas intraplate earthquake swarms that are not connected to active volcanism are present in continental rifts, such as the Rio Grande Rift, the Kenya Rift, and the western Eger Rift (Ibs-von Seht et al., 2008). Due to magmatic activity beneath the Cheb Basin, large-scale degassing of mantle-derived CO_2_ (>99%) occurs, and traces of gases such as He, N_2_, Ar, and CH_4_ are emitted (Weinlich et al., 1999; Kämpf et al., 2013; Hrubcová et al., 2017; Bräuer et al., 2018). The gas migrates through the upper lithospheric mantle and the crust to the surface and mixes with water of deep thermal and shallow groundwater aquifers (Bussert et al., 2017). Mofettes are local degassing phenomena that often occur as larger DDS on the scale of up to a few square kilometers. They are controlled by fluid migration in fault zones (Girault and Perrier, 2014; Nickschick et al., 2015) or in volcano-hydrothermal areas (Chiodini et al., 2008; Girault et al., 2014; Inguaggiato et al., 2017). The investigated dry mofette is part of the Hartoušov mofette field (HMF or DDS Hartoušov) according to Kämpf et al. (2013), which covers an area of approximately 350,000 m^2^ of grassland with two small ponds (2–6m^2^) close to the river Plesná. A total of 23–97 tons of CO_2_ have been estimated to be released daily in the HMF (Figure 1B; Nickschick et al., 2015).

The drilling sites were located along a NW-SE-oriented profile that is perpendicular to one of the main degassing areas of the HMF (Figure 1B). The CO_2_ soil gas flux was repeatedly measured in June, August, September, and October 2012 along a 55-m profile that consisted of 11 stations (P1 to P11) with 5 m between each of them (Nickschick et al., 2015). The CO_2_ soil gas flux measurements were performed by the accumulation chamber method, which uses a LiCOR 820 infrared gas analyzer for CO_2_ discharge quantification and two accumulation chambers (West Systems, Italy). The profile encompasses low, medium, and high CO_2_ soil degassing spots at the soil surface with strong degassing in the center of the profile (Nickschick et al., 2015). Places of high CO_2_ soil gas flux form small hummocks (Flechsig et al., 2008).

Two 3-m cores were retrieved by hammered drilling using a motor-driven hammer (Wacker Neuson, Germany). The cores were taken from a dry mofette near station P6 and an undisturbed reference site in the direct vicinity of station P2 (Nickschick et al., 2015; Supplementary Table S1 and Figures 1C,D). The mean CO_2_ soil gas flux of the mofette amounted to 27,961.6 g m^-2^ per day, while the CO_2_ soil gas flux of the reference site amounted to 8.1 g m^-2^ per day (Nickschick et al., 2015). At the mofette site, which has a size of about 2 m^2^, the growth of vegetation is hindered by continual CO_2_ degassing (Figure 1C). A pond about 4 m away from the sampled dry mofette is irregularly filled with groundwater or meteoric water (Supplementary Figure S1).

Each core was subsampled in technical triplicates in the topmost 1 m in intervals of 5 cm and below in intervals of 10 cm. The inner part of the core material was subsampled using an ethanol flamed spatula. The core material for molecular biological analyses was immediately stored at -20°C after subsampling.

### Geochemical Analysis

Pore water content in the sediment samples was too low to gain a sufficient amount of water for ion chromatographic analyses; therefore, a leaching procedure was applied in accordance with (Blume et al., 2011). Five grams of sediment were suspended in 25 mL of freshly autoclaved deionized water, shaken for 90 min in an anaerobic workstation (Don Whitley Scientific Limited, West Yorkshire, United Kingdom), and then centrifuged using airtight centrifuge tubes to remove solids. The water content of the fresh sediment was calculated from the difference in weight after being dried at 75°C for 2 days. The pH and the conductivity of the pore water were analyzed with a Multi 3420 SET G digital measuring instrument (WTW, Weilheim, Germany).

Total organic carbon (TOC) and δC_org_ values were measured using an elemental analyzer (NC 2500 Carlo Erba) coupled with a ConFlowIII interface on a DeltaPlusXL mass spectrometer (Thermo Fischer Scientific). Around 3 mg of sample material were weighed in unfolded Ag-capsules, added with 20% HCl, heated for 3 h at 75°C, enfolded in the Ag-capsules, and measured. The calibration of δC_org_ was performed by certified isotope standards (USGS24, CH-7) and proofed by an internal soil reference sample (Boden3). The isotopic composition is given in δC_org_ notation relative to a standard: δ(‰) = [(*R*_sample_–*R*_standard_)/*R*_standard_] × 1000. The ratio (R) and standard for carbon is ^13^C/^12^C and VPDB (Vienna PeeDee Belemnite).

The cation and anion concentrations in leached pore water were analyzed using ion chromatography (IC) (Sykam Chromatography, Eresing, Germany) according to Vuillemin et al. (2016) protocol. For cations, the IC system consisted of a S5300 sample injector (Sykam), a 4.6 mm × 200 mm ReproSil CAT column (Dr. Maisch HPLC, Ammerbuch-Entringen, Germany), and a S3115 conductivity detector (Sykam). The eluent was 5 mM H_2_SO_4_, and the eluent flow rate was set at 1 mL min^-1^. The column oven temperature was 45°C. A Cation Multi-Element IC-standard (Carl Roth) was diluted ten times for calibration. Samples and standards were measured in technical triplicates. For anions, the suppressed IC system consisted of a SeQuant SAMS anion IC suppressor (EMD Millipore, Billerica, MA, United States), a S5200 sample injector, a 3.0 mm × 250 mm lithocholic acid (LCA) 14 column, and a S3115 conductivity detector (all Sykam). The eluent was 5 mM Na_2_CO_3_ with 20 mg L^-1^ 4-hydroxybenzonitrile and 0.2% methanol. The eluent flow rate was set at 1 mL min^-1^, and the column oven temperature was set at 50°C. A multi-element anion standard (Sykam) was diluted ten times for calibration. Samples and standards were measured in technical triplicates.

### DNA Extraction and Purification

The total genomic DNA was isolated by the FastDNA^TM^ SPIN Kit for soil and the FastPrep^®^Instrument (MP Biomedicals, Santa Ana, CA, United States) with some protocol modifications. The FastPrep^®^ Instrument homogenizing time was set to 30 s, and the speed was set to 5.5 m s^-1^. The mixing time of Binding Matrix and DNA crude extract solution was extended to 20 min. The Genomic DNA Clean & Concentrator^TM^-10 (Zymo Research, Irvine, CA, United States) was utilized to remove humic acids and other substances that may have inhibited the PCR reaction. Three DNA isolations were extracted from each of the 44 sediment samples (22 samples from each core) as technical triplicates. In total, 132 samples were processed.

### Quantitative PCR

The total bacterial abundance (16S rRNA gene) and the functional genes of sulfate-reducing bacteria (SRB) (*dsrB* gene) and methanogenic archaea (*mcrA* gene) were determined by a quantitative polymerase chain reaction (qPCR). The qPCR Master Mix consisted of 12.5 μl iTaq^TM^ Universal SYBR^®^ Green Supermix (Thermo Fisher Scientific Inc., United States), 8.5 μl PCR water, 0.5 μl forward primer (20 μM), 0.5 μl reverse primer (20 μM), and 3 μl template. The quantification of the bacterial 16S rRNA gene was based on the primer pair of 331F (5′-TCCTACGGGAGGCAG-CAGT-3′) and 797R (5′-GGACTACCAGGGTATCTAATCCTGTT-3′) (Lane, 1991) and followed the protocol of 5 min at 98°C, 40 cycles of 5 s at 98°C, 20 s at 57°C, and 60 s at 72°C. The cloned 16S rRNA gene fragment from *E. coli* was used as standard. The qPCR efficiency for the 16S rRNA gene quantification was 90.2% and the *R*^2^-value of the standard curve line was 0.996. The quantification of the *dsrB* gene was based on the primer pair of dsr2060F (5′-CAACATCGTYCAYACCCAGGG-3′) and dsr4R (5′-GTGTAGCAGTTACCGCA-3′) (Ben-Dov et al., 2007) and followed the protocol of 10 min at 95°C, 40 cycles of 30 s at 95°C, 60 s at 60°C, 60 s at 72°C. The cloned *dsrB* gene fragment of *Desulfovibrio vulgaris* was used as standard. The qPCR efficiency for the *dsrB* gene quantification was 93.4% and the *R*^2^-value of the standard curve line was 0.999. The quantification of the *mcrA* gene was based on the primer pair of mlas-F (5′-GGTGGTGTMGGDTTCACMCARTA-3′) and mcrA-R (5′-CGTTCATBGCGTAGTTVGGRTAGT-3′) (Steinberg and Regan, 2009) and followed the protocol of 3 min at 95°C, 40 cycles of 5 s at 95°C, 20 s at 58.5°C, 30 s at 72°C, and 3 s at 80°C. The cloned *mcrA* gene fragment of *Methanosarcina barkeri* was used as standard. The qPCR efficiency for the *mcrA* gene quantification was 97.7% and the *R*^2^-value of the standard curve line was 0.997.

The qPCR was conducted on a CFX96 real-time thermal cycler (Bio-Rad Laboratories Inc., United States), and the analysis of the quantification data was performed with the CFX Manager^TM^ software (Bio-Rad Laboratories Inc., United States).

### Illumina MiSeq Amplicon Sequencing

The 16S rRNA gene amplified from extracted total genomic DNA was used as a template for the Illumina MiSeq high-throughput sequencing. The PCR reaction solution consisted of 12.5 μl MangoMix^TM^ (Bioline, Taunton, United States), 9.2 μl PCR water, 0.3 μl bovine serum albumin, 0.25 μl forward primer (20 μM), 0.25 μl reverse primer (20 μM), and 2.5 μl template. Unique combinations of barcode-tagged 515F (5′-GTGCCAGCMGCCGCGGTAA-3′) and 806R (5′-GGACTACHVGGGTWTCTAAT-3′) (Caporaso et al., 2011) primers were assigned to each sample. The amplifications were performed on a T100 thermal cycler (Bio-Rad Laboratories Inc., United States) and followed the protocol of 3 min at 95°C, 30 cycles of 30 s at 94°C, 45 s at 56°C, 60 s at 72°C, and a final extension step of 10 min at 72°C. The PCR products were cleaned up with AMPure XP magnetic beads (Beckman Coulter GmbH, Krefeld, Germany). After measuring the DNA concentration (CLARIO star^®^ plate reader, BMG LABTECH GmbH, Ortenberg, Germany), PCR products were pooled in equimolar amounts. The DNA pool was concentrated (Eppendorf Concentrator plus, Eppendorf AG, Hamburg, Germany) to meet the requirement of sequencing (DNA concentration ≥50 ng μl^-1^). It should be noted that the used MangoMix^TM^ (Bioline, Taunton, United States) does not supply a proof-reading polymerase, which may inflate species richness and interfere with recovery of certain genotypes (Brandariz-Fontes et al., 2015). However, Pereira et al. (2018) showed that the choice of DNA polymerase did not significantly change the community profiling and composition.

### Bioinformatics and Statistical Analysis

Sequencing was performed by Eurofins Scientific SE, Luxembourg, on an Illumina MiSeq (2 × 250 bp). Read pairs were merged using PEAR (Zhang et al., 2014). QIIME (Version 1.9.1; Caporaso et al., 2010) was employed for microbiome analysis. More specifically, reads were demultiplexed, and USEARCH (Edgar, 2010) was used for the detection and removal of chimeric sequences. The SILVA database (Version 128; DeSantis et al., 2006) was utilized for open-reference OTU clustering (97% sequence similarity) and taxonomic assignments. Rational taxonomic boundaries have been proposed for the high taxa (that is, genus and above) of the Bacteria and the Archaea on the basis of 16S rRNA gene sequence identities. These are: 94.5% for genus, 86.5% for family, 82.0% for order, 78.5% for class, and 75.0% for phylum (Yarza et al., 2014). Within in this study the taxonomical data was discussed on genus-level and above. Singletons and OTUs assigned to chloroplasts were removed. The data received for the technical triplicates were merged for the downstream analyses. For alpha diversity analyses, the data were rarefied to 14,528 reads per sample. Alpha diversity and evenness were analyzed using the Shannon H index and the Shannon EH index. Beta diversity (PCoA) was determined by calculating the weighted UniFrac distance metric (QIIME), and samples from different depths were clustered and illustrated by 95% confidence ellipses. For multivariate statistics (including canonical correlation analysis, CCA), CANOCO 5 (Šmilauer and Lepš, 2014) and PAST3 (Hammer et al., 2001) were used. Sequencing data were submitted to the European Nucleotide Archive^1^ under accession numbers ERS 2039641 to ERS 2039772 (Bioproject PRJEB22478).

## Results

### Stratigraphy and Geochemical Characterization

**FIGURE 2:**
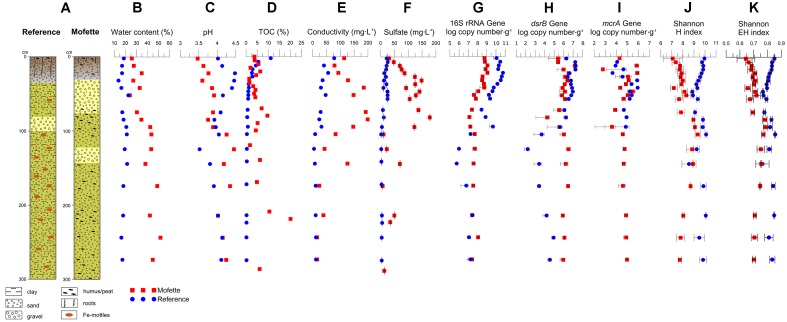
Lithological profile of the reference and mofette core **(A)**. The water content **(B)**, pH **(C)**, TOC **(D)**, conductivity **(E)**, sulfate concentration **(F)**, bacterial 16s rRNA gene copy number **(G)**, *dsrB* gene copy number **(H)**, *mcrA* gene copy number **(I)**, Shannon H index **(J)** and Shannon EH index **(K)** of reference (blue dots) and mofette (red squares) core.

The reference core consisted primarily of fine- to medium-grained clayey sand that contained dispersed iron mottles (Figure 2A). A gravel-rich sand layer was reached at a depth of 80 to 100 cm, which roughly represented the groundwater level. The mofette core was dominated by humus or peaty sand and occasional by sandy peat. Clay or clayey sand occurred primarily in the topmost 20 cm. In the mofette field, the groundwater level was shallower (25–70 cm) than at the reference site. The very shallow groundwater level was also reflected in the high water content of the sediment (Figure 2B).

Ion concentrations, conductivity, and pH were obtained by sediment leaching (Figures 2C,E,F). The complete spectrum of measured anions, cations, and δC_org_ values is provided in the Supplementary Tables S1, S2. The concentrations of sulfur, nitrate, and nitrite were under detection limit. The pH values in the mofette core increased from around 3.5–4.3 down to a depth of 105 cm and varied from 4.0 to 4.6 between 105 and 275 cm in depth. In contrast, the pH values of the reference core ranged from 3.5 to 4.5 without displaying a clear trend. The conductivity of the mofette sediment was substantially higher compared with the reference core. Conductivity in the mofette site increased from 114 mg L^-1^ at the surface to 199 mg L^-1^ at a depth of 85 cm and decreased downward to 16 mg L^-1^ at a depth of 275 cm. A conductivity peak of 126 mg L^-1^ was measured at a depth of 145 cm. In the reference core, the conductivity decreased from top to bottom from 78 mg L^-1^ to 12 mg L^-1^.

The TOC content of the reference site decreased from 11.1% in the topsoil to 0.2% in the deepest sample at 275 cm in depth. The mofette site was characterized by a generally higher TOC content that varied significantly in the organic-rich peat layers (Figure 2D). The highest TOC contents were present at a depth of 224 cm (20.1%) and below the groundwater table at a depth of 82 cm (9.6%). δC_org_ values were in the range of -25.3 to -28.5‰, and showed no significant differences between mofette and reference site.

In comparison with the reference core, sulfate concentrations were higher across the entire mofette core (Figure 2F). The sulfate most likely stems primarily from the oxidation of pyrite, which at Hartoušov (Flechsig et al., 2008) proved to be abundant in the mofette sites. The highest sulfate concentrations were measured in the top 65 cm of the reference (up to 25.74 mg L^-1^) and in the top 100 cm of the mofette core (up to 177.92 mg L^-1^). Sulfate concentrations were ten times higher than other measured anion and cation concentrations in the mofette and had a strong positive correlation with conductivity (*R* = 0.8019, *p* < 10^-5^). Therefore, the high sulfate concentrations seem to be the main reason for the high conductivity.

### Abundance of Microorganisms

The bacterial abundance (16S rRNA gene copy numbers) in the mofette core decreased within the upper 50 cm (Figure 2G). However, no decrease in abundance was observed below 50 cm in sediment depth. From 0 to 100 cm in sediment depth, the gene copy numbers of the reference core were one order of magnitude higher than those of the mofette core. The highest bacterial abundance was analyzed at a depth of 12 cm in the mofette core (5.8 × 10^10^ gene copies g^-1^ sediment) and at 22 cm in the reference core (1.2 × 10^9^ gene copies g^-1^ sediment). From 100 to 280 cm in sediment depth, the gene copy numbers in the mofette core partially exceeded those of the reference site.

The abundance of SRB was estimated through the quantification of *dsrB* gene copies (Figure 2H). The *dsrB* gene copy numbers in the reference core followed a similar trend to that of the respective 16S rRNA gene copy numbers. The highest *dsrB* gene copy number (1.9 × 10^7^ gene copies g^-1^ sediment) was measured at a depth of 12 cm, and the lowest (1.6 × 10^2^ gene copies g^-1^ sediment) was measured at 125 cm. The *dsrB* gene copy numbers in the mofette core decreased with depth (1.5 × 10^7^ to 1.8 × 10^4^ gene copies g^-1^ sediment).

The abundance of methanogens was estimated via the quantification of *mcrA* gene copies (Figure 2I). Between a depth of 0 and 100 cm, the *mcrA* gene copy numbers of both the mofette site and the reference site varied in the range of 0 and 8.5 × 10^5^ gene copies g^-1^ of sediment. No *mcrA* genes were detected at depths greater than 100 cm at the reference site; however, *mcrA* gene copy numbers in the mofette site slightly increased with depth (3.9 × 10^4^ gene copies g^-1^ sediment at a depth of 105 cm to 9.3 × 10^4^ gene copies g^-1^ sediment at a depth of 275 cm).

### Community Structure

In total 10,201,992 sequences were obtained in the 16S rRNA gene library after merging, demultiplexing, filtering, and excluding of chimeric sequences, chloroplast-like sequences, and singletons. The read numbers ranged from between 21,143 and 160,896, with a mean value of 77,287 (Supplementary Table S3). Rarefaction analyses revealed that no sample exhibited a conspicuous increase in its Shannon H index when calculating more than 14,528 sequences per sample (Supplementary Table S4).

Except for the section of sediment between 85 and 145 cm in depth, the reference site showed a higher alpha diversity (Figure 2J and Supplementary Table S4). The alpha diversities of the mofette increased with depth from 0 to 85 cm, remained constant from 85 cm to 145 cm, and then decreased toward the end of the profile. The Shannon index of the mofette displayed a weak positive correlation with pH (*R* = 0.582, *p* = 0.0045) and water content (*R* = 0.497, *p* = 0.0187) (Supplementary Tables S5, S6). The Shannon EH equitability index was lower throughout the depth sequence at the mofette than at the reference site (Figure 2K), especially at the surface layers and the deepest part.

**FIGURE 3:**
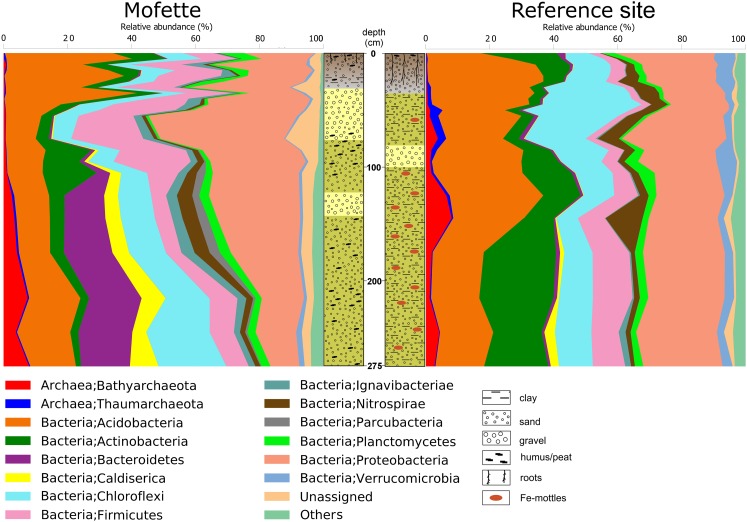
Community structures at phylum level (most abundant 14 phyla) and the lithological profiles of the cores.

The relative abundance of each taxon is displayed by the percentage of total sequence reads (Supplementary Table S7). The dominant phyla at both the mofette site and the reference site were *Acidobacteria* (mofette: 20.4%; reference: 25.6%), *Actinobacteria* (mofette: 6.7%; reference: 10.9%), *Chloroflexi* (mofette: 9.4%; reference: 15.8%), *Firmicutes* (mofette: 14.9%; reference: 4.8%), and *Proteobacteria* (mofette: 24.9%; reference: 23.7%). The dominant archaeal phyla were *Bathyarchaeota* (mofette: 1.8%; reference: 1.7%), *Euryarchaeota* (mofette: <0.1%; reference: 0.4%), and *Thaumarchaeota* (mofette: 0.3%; reference: 1.0%). Moreover, a larger fraction of unassigned taxa were present in the mofette sediments (4.0%) compared with in the reference sediments (1.0%; Figure 3).

**FIGURE 4:**
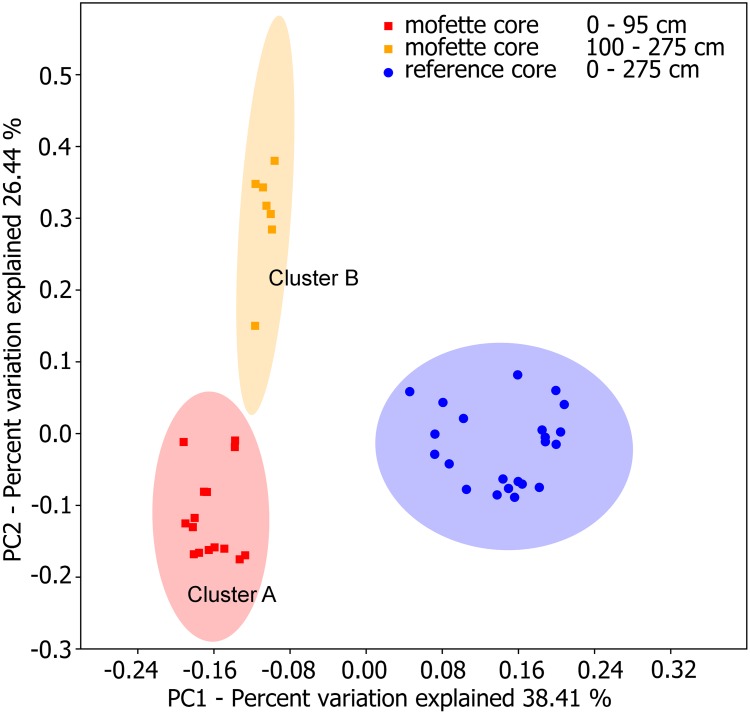
PCoA plot calculated by the weighted UniFrac distance of the microbial communities. Mofette samples are indicated by red and orange squares; reference site samples by blue dots. The 95% confidence ellipses indicate three different clusters.

Beta diversities were obtained by calculating a weighted UniFrac distance metric (Figure 4). A distinct clustering of the microbial communities was observed for both sites. The microbial community structure of the upper sediment (0–95 cm) of the mofette was distinct from the deeper part of the core (100–275 cm), and we therefore defined two distinct clusters. Cluster A includes communities from 0 to 95 cm in depth, and Cluster B includes communities observed at depths between 100 and 275 cm.

**FIGURE 5:**
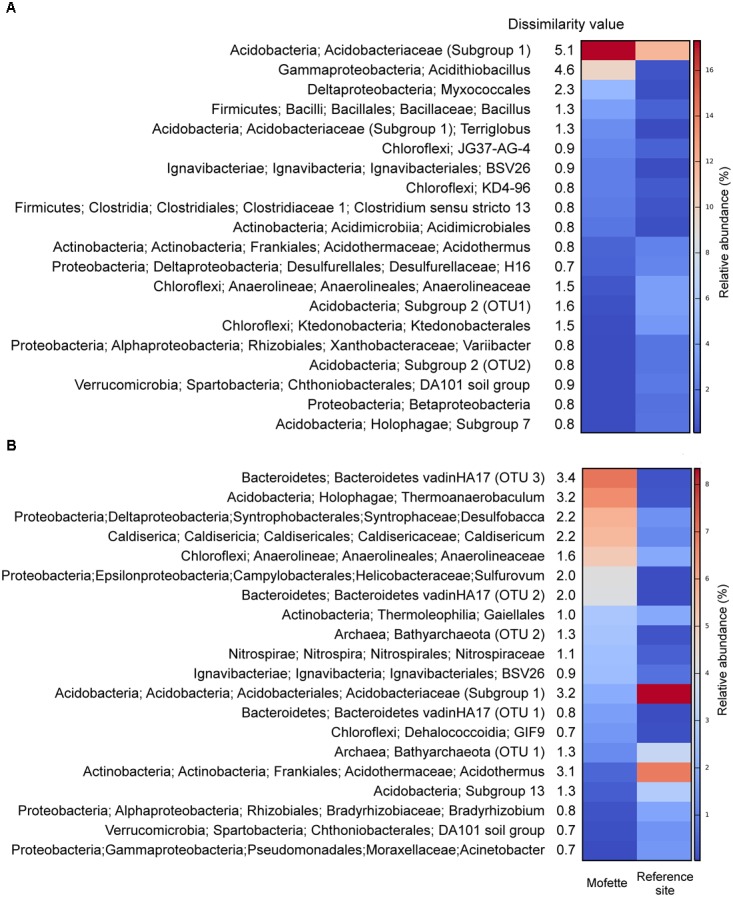
Heatmap of the top 20 taxa which explain most of the dissimilarity between the microbial communities of the mofette and the reference site based on the Bray–Curtis dissimilarity values. Cluster A (depths between 0 and 95 cm) **(A)** and Cluster B (depths between 100 and 275 cm) **(B)**.

At a depth of 0–100 cm (Cluster A; Supplementary Table S8), the dominant taxa were *Acidobacteriaceae* (Subgroup 1) (mofette: 12.4%; reference: 10.5%) – which contributed to 5.1% of the dissimilarity – followed by the genera *Acidithiobacillus* (mofette: 7.1%; reference: 0.5%) – which contributed to 4.6% of the dissimilarity (Figure 5A). At a depth of 100–275 cm (Cluster B; Supplementary Table S9), where the influence of the uprising CO_2_ was stronger than in the atmospheric- and groundwater-influenced upper part of the depth sequence, significant differences in the community structures were observed (Figure 3). *Acidothermus* (mofette: 0.5%; reference: 6.8%) and *Acidobacteriaceae* (Subgroup 1) (mofette: 2.0%; reference: 8.3%) were much more abundant in the reference site, and *Sulfurovum* (mofette: 4.2%; reference: 0.1%), *Anaerolineaceae* (mofette: 5.1%; reference: 2.0%), *Caldisericum* (mofette: 5.5%; reference: 1.2%), *Desulfobacca* (mofette: 5.7%; reference: 1.4%), *Thermoanaerobaculum* (mofette: 6.5%; reference: 0.3%), and *Bacteroidetes vadin HA17* (mofette: 4.1%; reference: 0.1%) were more abundant in the mofette (Figure 5B).

**FIGURE 6:**
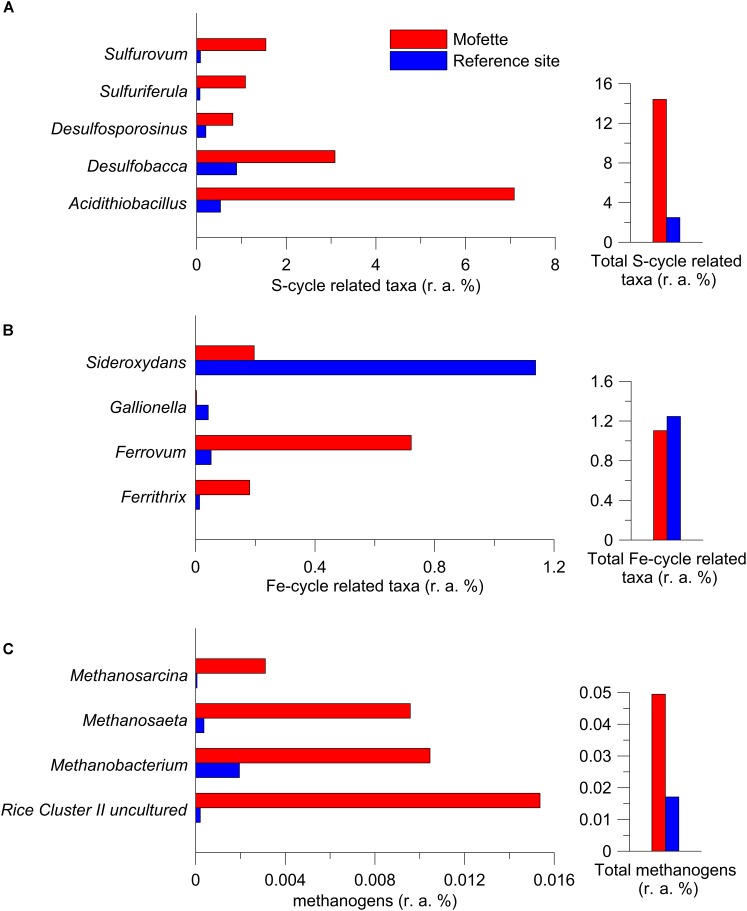
Relative abundance (r. a.) of the most abundant taxa and the total fraction involved in the sulfur-cycle **(A)**, iron-cycle **(B),** and methanogenesis **(C)**.

In the mofette site, microorganisms potentially involved in sulfur cycling were far more abundant (relative to the entire community) and comprised up to 14.4% of the sequence reads. In contrast, only 2.5% of the sequence reads were involved in sulfur cycling at the reference site. Observed taxa involved in sulfur cycling were sulfide/sulfur oxidizer *Acidithiobacillus* (mofette: 7.1%; reference: 0.5%) (Kelly and Wood, 2000), *Sulfuriferula* (mofette: 1.1%; reference: 0.1%) (Watanabe et al., 2015), *Sulfurovum* (mofette: 1.5%; reference: 0.1%) (Inagaki et al., 2004), SRB *Desulfobacca* (mofette: 3.1%; reference: 0.9%) (Oude Elferink et al., 1999), and *Desulfosporosinus* (mofette: 0.8%; reference: 0.2%) (Stackebrandt et al., 1997; Figure 6A). Taxa involved in iron cycling were less abundant in the mofette (1.1%) than at the reference site (1.2%). The iron cycling-related taxa consisted of extremely acidophilic iron-oxidizer *Ferrithrix* (mofette: 0.18%; reference: 0.01%) (Johnson et al., 2009), acidophilic iron-oxidizer *Ferrovum* (mofette: 0.72%; reference: 0.05%) (Johnson et al., 2014), *Gallionella* (mofette: <0.01%; reference: 0.04%) (Hallbeck and Pedersen, 1990), and the aerobic iron-oxidizer *Sideroxydans* (mofette: 0.20%; reference: 1.14%) (Emerson and Moyer, 1997; Figure 6B). Methanogens were found in a relatively low proportion at both the mofette site and the reference site (0.04 and 0.02%). Uncultured taxa from Rice Cluster II (Großkopf et al., 1998), *Methanobacterium* (Smith et al., 1997), *Methanosaeta* (Patel and Sprott, 1990), and *Methanosarcina* (Zinder et al., 1985) were the most abundant genera and were proportionally more common in the mofette microbial community (0.02, 0.01, 0.01, and <0.01%) in comparison with those of the reference site (<0.01%; Figure 6C).

The mofette and the reference site shared 1,626 taxa, and a small fraction of taxa only occurred in the mofette (138) or the reference site (128). At depths of between 100 and 275 cm, 1,336 taxa were shared, but 184 and 198 taxa were only observed in the mofette and at the reference site, respectively. A total of 1,045 taxa were detected in the deeper part (200–275 cm) of the mofette and the reference site, 124 taxa were detected only at the mofette, and 330 taxa were only detected at the reference site (Supplementary Table S10).

Interestingly, methanogenic archaea – such as *Methanosphaerula* – were only found in the mofette. *Methanoregula* was not observed at the reference site between depths of 100–275 cm. *Methanosaeta* and *Methanosarcina* were not observed in the deep layers (200–275 cm) of the reference site.

### Multivariate Statistics

**FIGURE 7:**
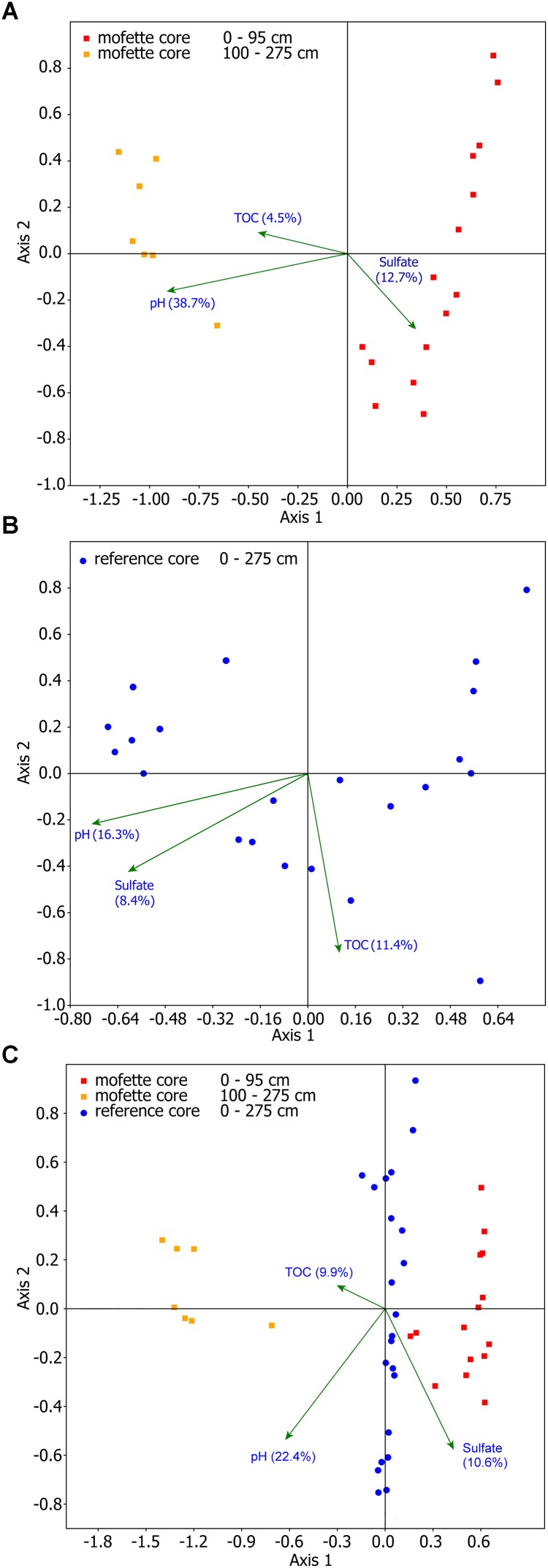
CCA analyses of the microbial communities of the mofette **(A)**, reference site **(B)**, and for both **(C)**. The environmental parameters were selected based on the forward-selection method with significant *p*_adj_-values (*Bonferroni* corrected <0.05).

Canonical-correlation analysis was used to determine community-shaping environmental factors (Figure 7). Among all measured environmental parameters, only those with significant *p*_adj_-values (*Bonferroni* corrected <0.05) were included in the analyses (Supplementary Table S11). pH, sulfate concentration, and TOC formed the optimal subset of parameters to explain the observed OTU distribution. The pH value explains 38.7% of the distribution pattern of taxa at the mofette site (Figure 7A) and 16.3% at the reference site (Figure 7B). Sulfate concentration explains 12.7% of the distribution pattern of microorganisms at the mofette (Figure 7A) and 8.4% at the reference site (Figure 7B). The TOC values explain 11.4% of the distribution pattern at the reference site (Figure 7B) and 4.5% at the mofette (Figure 7A). The differences in the community structure of the mofette and the reference site are mainly explained by pH (22.4%), sulfate concentration (10.6%), and TOC (9.9%) (Figure 7C).

The 16S rRNA gene copy numbers at the reference site are positively correlated with pH (*R* = 0.709, *p* = 0.0002), whereas at the mofette site, a significant negative correlation with pH (*R* = -0.655, *p* = 0.0009) was observed (Figure 2G) (Supplementary Tables S5, S6). No significant correlation with any measured environmental parameter was found for the abundance of the *dsrB* or *mcrA* genes.

## Discussion

Dry mofettes – such as the HMF – allow for an investigation of geo–bio interactions that result from the permanent degassing of mantle-derived CO_2_. In the uppermost soil layers – which have been the focus of other studies (Krüger et al., 2009; Blagodatskaya et al., 2010; Sáenz de Miera et al., 2014; Beulig et al., 2015) – the conditions are not necessarily permanent strictly anoxic, which also allows aerobic or microaerophilic heterotrophic microorganisms to grow. The input of oxygen can derive from meteoric water as well as horizontally via groundwater flow. The sampling depth and lithological profile are therefore of major importance in unraveling the influence of the CO_2_ on the microbial communities. For the first time, the present study provides insights into the community structure in 3-m-deep sediments of a mofette and a nearby reference site. The combination of geochemical analyses and Illumina MiSeq high-throughput sequencing of 16S rRNA genes reveals the complexity of geo–bio interactions in the CO_2_-influenced habitat.

We observed a strong influence of the emanating CO_2_-rich fluid on crucial soil parameters, such as pH, water content, and ion composition (Figure 2). The relatively low pH may be an indication for the influence of CO_2_ also at the reference site, and reflects the characteristics of the lithological profile. However, the CO_2_ flux measurements at the surface of the reference site did not reveal any mofette activity; this is supported by the typical TOC profile and low sulfate concentration and conductivity. Both sampling sites were located in a wet land area (floodplain) with a high groundwater level. We assume that depending on the season (and flooding) an influence by CO_2_-rich groundwater at both sites is possible. It is known that the groundwater level at the HMF may shift around 20 cm per day (Nickschick et al., 2017). Within the first 80 cm of the depth profile the pH of the reference site (pH > 4) was higher than at the mofette site (pH < 4). At the reference site the groundwater level is situated within a gravel-rich sand layer at a depth of 80 to 100 cm. The groundwater, which may migrate horizontally, presumably causes a decrease of the pH due to dissolved CO_2_. Thereby, the fine- to medium-grained clayey sand in the reference site might function as a natural vertical barrier for the groundwater and CO_2_ flow, especially in depths deeper than the core sequence. In the mofette, the groundwater level was shallower (25–70 cm) than at the reference site. On the other hand the pH in the mofette was highest in the gravel-rich layer between depths of 130–140 cm. The high conductivity and sulfate concentration (Figure 2) may indicate that mineral water from greater depth is admixed with groundwater in the mofette. Mineral water found in the same mofette in a depth of ca. 82 m had a pH of 6.4 and a sulfate concentration 1470 mg L^-1^ (Bussert et al., 2017).

The different microbial community structures found in each depth and each core at the reference site and in the mofette cannot be explained by the variances in the lithology of the core material, however, the lithological setting is from a major importance for both habitats in greater depth (78 m) where a cap-rock like carbonate-rich layer largely seals the CO_2_-rich aquifer and allows for a channelized CO_2_ degassing (Bussert et al., 2017). Instead, the pH, TOC, and sulfate concentration formed the optimal subset of parameters to explain the abundance and distribution of the taxa at both sites. Multivariate statistical analyses and qPCR results revealed that at the reference site, the microbial abundance was positively correlated with pH (*R* = 0.709, *p* = 0.0002), while a significant negative correlation was observed at the mofette site (*R* = -0.655, *p* = 0.0009; Figures 2C,G and Supplementary Tables S5, S6). Therefore, several taxa of the microbial community at the mofette site seem to be adapted to the acidic conditions. However, the diversity and the evenness at the mofette site are positively correlated with the pH (*R* = 0.582, *p* = 0.0044, and *R* = 0.550, *p* = 0.008; Supplementary Tables S5, S6), indicating that it is mainly specialists that can withstand the extreme environmental conditions. It is remarkable that solely the pH value explains 38.7% of the distribution pattern of taxa at the mofette site (Figure 7A). Since the pH value at the reference site is hardly affected by the emanating CO_2_, it only explains 16.3% of the OTU distribution (Figure 7B). However, since the pH is generally co-correlated with CO_2_ concentration, it is necessary to consider the fact that the abundance of autotrophic microorganisms in the mofette site is also positively correlated with the amount of CO_2_-rich emanating fluids. The results of the geochemical analyses and the 16S sequencing lead to the assumption that in addition to pH, sulfate also plays an important role in microbial matter cycling under anaerobic conditions in the mofette site. The sulfate concentration – which is five to fifteen times higher in the mofette site – explains 12.7% of the distribution pattern of microorganisms at the mofette (Figure 7A). In comparison with the mofette, the sulfate concentration at the reference site explains only 8.4% of the community structure (Figure 7B).

In comparison with the reference site, the TOC values were up to 100 times higher in the peat layers of the mofette (Figures 2A,D). While pH and ion concentrations are directly influenced by the emanating CO_2_, the high TOC content at the mofette site can be explained by the consequences of acidification and anoxia on mesoscopic or macroscopic eukaryotes involved in the degradation of complex organic matter; additionally it was shown that acetogenesis is a prominent process in mofettes (Beaubien et al., 2008; Beulig et al., 2015, 2016; Fernández-Montiel et al., 2016). The TOC values explain 11.4% of the distribution pattern at the reference site (Figure 7B) and only 4.5% at the mofette (Figure 7A). Moreover, the top 10 abundant taxa at the reference site were heterotrophic microorganisms (Supplementary Tables S8, S9). In contrast, the high abundance of chemolithoautotrophic taxa in the mofette highlights the importance of an autotrophic- rather than a heterotrophic lifestyle in habitats with strongly elevated CO_2_ concentrations (Oppermann et al., 2010). Autotrophic microorganisms can fix significant amounts of carbon from geogenic CO_2_ (Nowak et al., 2015), whereas organic substrates remain undegraded and accumulate in the sediment. An interesting side effect can be seen in a long-term perspective because such an enormous accumulation of organic substances in sediments may be the precondition for the development of a paleo organic layer at a late stage.

The bacterial 16S rRNA gene copy numbers in the mofette varied from 10^5^ to 10^9^ copies g^-1^ of sediment. The highest gene copy numbers were found in the topsoil of the mofette (1.2 × 10^9^ gene copies g^-1^ at a depth of 12 cm; Figure 2G). While the abundance of microorganisms at the reference site followed a classical trend and decreased with depth, the abundance of microorganisms in the mofette did not decrease with depth beyond 50 cm. The present study suggests for the first time that at a depth greater than 100 cm, the microbial abundance in the mofette even partially exceeded that of the reference site. This trend was also observed for SRB and methanogenic archaea (Figures 2H,I). As discussed later, presumably the uprising CO_2_-rich fluid feeds the ecosystem from underneath with substrates such as CO_2_ and sulfate. However, compared with the reference site, the gene copy numbers at the mofette were about one order of magnitude lower for the uppermost 100 cm. Higher cell numbers in the surface soil of the reference site can be explained by the predominance of aerobic processes and a rather moderate pH. In general, the gene copy numbers per gram of subsurface sediment at the HMF were similar to soils with highly elevated CO_2_ concentrations (10^9^ to 10^10^ copies g^-1^ sediment) (Beaubien et al., 2008; Krüger et al., 2009; Oppermann et al., 2010; Frerichs et al., 2013; Beulig et al., 2015; Fernández-Montiel et al., 2016). The top 40 cm of the mofette site at the HMF was an exception, for here, the gene copies were one order of magnitude lower (10^8^ to 10^9^ copies g^-1^ sediment) compared with the Plesná floodplain site, which is located 1.8 km to the NW (Beulig et al., 2015). Reasons for the discrepancy could be the higher CO_2_ flux at the HMF or different soil characteristics (Kämpf et al., 2013). Additionally, seasonal effects have to be considered. In April (Beulig et al., 2015), the surface water level is often higher, and substrates might be more easily accessible in comparison with September, when the HMF drilling took place.

Both diversity and evenness were lower at the mofette than at the reference site (Figures 2J,K). A lowered diversity was also observed at the La Sima mofette in Spain (Sáenz de Miera et al., 2014). The low evenness indicates a dominance of specialists, such as anaerobic acidophilic and acido-tolerant taxa in the mofette community. In the mofette, the alpha diversity and the evenness were higher at depths between 85 and 125 cm. This shift can be explained by the surface water table and the admixture of surrounding aerobic communities.

The results of the beta diversity of the community highlight the assumption that only the deeper sediments are almost unaffected by oxygen. The PCoA plot of the weighted UniFrac distance metric revealed that the mofette harbors two distinct communities (Figure 4). One cluster includes communities from shallow depths between 0 and 95 cm (Cluster A), and the other cluster includes communities in the deep anoxic sediments from 100 to 275 cm (Cluster B). The microbial communities from the reference site cluster apart from all mofette samples, which highlights the fact that the mofette community is strongly influenced by the degassing phenomenon. Site-specific effects – such as a low pH and changes in ion composition – do not sufficiently explain the differences between Cluster A and Cluster B, but the oxygen availability could be identified as the major stress factor. In the mofette, the oxygen-dependent depth gradient was clearly reflected in the antagonistic shift of the relative abundance of aerobic and anaerobic taxa in Cluster A and Cluster B. The relative abundance of obligate and facultative anaerobic microorganisms affiliated with *Bacteroidetes*, *Bathyarchaeota*, *Caldiserica*, and *Parcubacteria* gradually increased with depth. At the same time, the relative abundance of versatile *Proteobacteria* strongly decreased with increasing depth (Figure 3). Strictly anaerobic and facultative aerobic microorganisms affiliated with *Acidithiobacillus*, *Clostridiaceae* 1, *Bacillus*, and *Ignavibacteriales* that were observed in Cluster A were much more abundant at the mofette than at the reference site. In Cluster B, strictly anaerobic microorganisms within the taxa *Thermoanaerobaculum*, *Bacteroidetes vadin HA17*, *Desulfobacca*, *Caldisericum*, *Anaerolineaceae*, and *Sulfurovum* shaped the community and explained most of the differences (14.5% dissimilarity) between the communities of the deep sediments of the mofette and reference site (Figures 5A,B). Neither in Cluster A nor Cluster B were any of the strictly aerobic taxa more abundant in the mofette than at the reference site, except for *Acidobacteriaceae* (Subg. 1). Additionally, the family *Acidobacteriaceae* (Subg. 1) was the most abundant taxa in both study sites (mofette: 12.4%; reference site: 10.5%). Members of this group are heterotrophic, aerobic, or microaerophilic, and some species are facultative anaerobes (Pankratov et al., 2012). Therefore, the occurrence at both sites under both aerobic and anaerobic conditions is plausible. The HMF multivariate statistics indicate a strong negative correlation of the taxon with pH, and the same correlation has been observed by Männistöet al. (2007) in tundra soils. Moreover, the proportion of unassigned taxa (4.0%) was much higher in the mofette compared with the reference site, indicating that mofettes are unique environments that harbor a large fraction of novel, as-of-yet undescribed organisms.

The sulfate-rich mofette with a pH < 4.0 offers ideal growth conditions for obligate acidophilic bacteria such as *Acidithiobacillus* (Kelly and Wood, 2000). Accordingly, these bacteria’s abundance is positively correlated with the sulfate concentration (*R* = 0.699, *p* = 0.0003; Supplementary Tables S5,S6). In both clusters, taxa potentially involved in sulfur cycling had a more substantial proportion (14.4%) and a higher diversity (e.g., of *Desulfobacca*, *Desulfosporosinus*, *Sulfurovum*, and *Sulfuriferula*) in the mofette in comparison with the reference site (3.0%; Figure 6A). *Desulfobacca* was also recently found in a Plesná floodplain site (Beulig et al., 2016). A higher abundance of SRB was also observed in CO_2_ vents (Beaubien et al., 2008) and CO_2_-affected soils (Frerichs et al., 2013). Striking differences between the mofette and reference site were also observed for taxa potentially involved in iron-cycling. The results lead to the assumption that in addition to sulfate reduction, iron-cycling is also an important feature under aerobic conditions at the reference site and under anaerobic conditions in the mofette. The aerobic or microaerophilic taxa – such as *Gallionella* and *Sideroxydans* (Hallbeck and Pedersen, 1990; Emerson and Moyer, 1997) – were more abundant at the reference site, whereby acidophilic obligate and facultative anaerobic genera – such as *Ferrithrix* and *Ferrovum* (Johnson et al., 2009; Johnson et al., 2014) – dominated in the mofette (Figure 7B).

The extreme environmental conditions in the mofette also favor the growth of methanogenic archaea. *Methanosphaerula* (Cadillo-Quiroz et al., 2009) occurred solely in the mofette, whereby other genera – such as *Methanoregula* (Bräuer et al., 2011), *Methanosaeta* (Patel and Sprott, 1990), and *Methanosarcina* (Zinder et al., 1985) – were abundant at all depths in the mofette core but occurred only in low abundances close to the surface in the reference site, presumably because oxygen is spatially depleted by aerobic processes. Other archaea potentially involved in acetogenesis and methane cycling – such as *Bathyarchaeota* and ammonia-oxidizing *Thaumarchaeota* affiliated with South African Gold Mine Gp 1 (SAGMCG-1) (Takai et al., 2001) – were found at the mofette and the reference site in almost the same relative abundances (1.7 and 0.2%). These organisms have been found in marine sediments, deep aquifer waters, a CO_2_ vent, as well as in water from wet mofettes and in thermal water in the Eger region (Kubo et al., 2012; Frerichs et al., 2013; Evans et al., 2015; Krauze et al., 2017).

Additionally, several other microorganisms found in the deep mofette sediments (e.g., *Thermoanaerobaculum*, *Caldisericum*, *Sulfurovum*, and *Mobilitalea*) have been isolated from hot springs, hydrothermal sediments, or thermal water from a 2.8-km-deep well (Oude Elferink et al., 1999; Inagaki et al., 2004; Losey et al., 2013; Podosokorskaya et al., 2014). These microorganisms most likely derive from the deep biosphere, indicating that the mofette is connected to the deep subsurface via ascending fluids. The environmental conditions in the mofette at 1–3 m in depth are similar to deep sediments or deeply originating thermal waters with respect to the availability of oxygen and ion composition and may therefore also provide adequate growth conditions for such taxa.

The microbial communities from both Cluster A and Cluster B at the HMF share many abundant taxa with the communities described by Sáenz de Miera et al. (2014) in the shallow sediments at the La Sima CO_2_ gas vent (top 10–20 cm; Supplementary Table S12) and a CO_2_-influenced floodplain site at Plesná (Beulig et al., 2015). Although the relative abundances of these common taxa display clear differences, they represent an outline of the characteristic core community of CO_2_-influenced surface habitats. The occurrence of unshared site-specific taxa can be explained by the differing environmental conditions. For example, higher oxygen concentrations (<7%) (Peinado et al., 2009) and historical thermal anomalies might specifically trigger the growth of aerobic or facultative anaerobic thermophiles – such as members of *Ktedonobacteria* (e.g., *Thermogemmatispora*) – at the La Sima mofette. The high resemblance of the Cluster A communities to the microbial communities at the CO_2_-influenced Plesná floodplain site (Beulig et al., 2015) lead to the assumption that the environmental conditions at both mofettes located in the Cheb Basin are rather similar compared with those the La Sima site. The low pH at the HMF and the floodplain particularly favors the growth of acidophilic taxa (e.g., *Acidobacteria*, Subg. 1), which have a rather low abundance at the La Sima site.

The high relative abundance of sulfate reducers, methanogens as well as further autotrophs indicate that the community is supported by hydrothermal originating substrates, delivered by the mantle-derived CO_2_. The geochemical data demonstrate that electron acceptors such as sulfate and CO_2_ are sufficient available. This is in good accordance with the findings from Beulig et al. (2016) who showed that in the mofette transcripts related to methanogenesis (mcr) and sulfate reduction (dsr, cys, apr) were remarkably increased in the frequency. In the Eger Rift, hydrogen, which is a key electron donor in the deep biosphere (Stevens and McKinley, 1995; Anderson et al., 2001; Chapelle et al., 2002; Nealson, 2005; Spear et al., 2005; Hinrichs et al., 2006), becomes available during radiolytic decay in the underlying fissured granite or stress-released during earthquake swarms (Bräuer et al., 2005, 2007).

## Conclusion

Our study of a dry CO_2_ degassing mofette in Hartoušov, NW Bohemia, as central part of a CO_2_ conduit deepens the knowledge of geo–bio interactions in extreme environments with elevated CO_2_ concentrations. The mofette ecosystem is characterized by anoxic conditions, a low pH, high TOC content and due to the admixing of mineral waters a relatively high sulfate concentration and conductivity. Our study shows that the exceptional environmental conditions provoke a decrease in diversity and favor the occurrence of anaerobic, acidophilic taxa, whereby sulfate reduction and methanogenesis become distinct processes. However, the deeper mofette sediments alone provide strictly anaerobic conditions, and accordingly, two distinct community clusters were found at different depth intervals. Electron acceptors such as CO_2_ and sulfate are provided by the permanently ascending fluid which is admixed with deep thermal waters, thereby forming a kind of anoxic deep biosphere habitat close to the surface level. Hydrogen, originating from deep fissured granites may thereby function as an electron donor. This study is limited to taxonomical assignments, further studies focusing on deep mofette sediments should implement metagenomic or transcriptomic approaches to unravel the functional repertoire of the communities.

## Author Contributions

All authors have taken part in the interpretation of the results and writing the manuscript. QL wrote the manuscript, performed sampling, pore water analyses, DNA extractions and purification, gene quantification, and bioinformatical based statistical analyses. HK and TN performed CO_2_ soil gas flux measurements. RB led the drilling campaign and performed sedimentological analyses. FH and PK were involved in statistical analyses. BP performed TOC and isotopic analyses. DW contributed to the interpretation of the results and valuable discussion. MA designed and supervised the study and led the writing of the present manuscript. All authors have taken part in the manuscript revisions and agreed with its scientific content.

## Conflict of Interest Statement

The authors declare that the research was conducted in the absence of any commercial or financial relationships that could be construed as a potential conflict of interest.

## Footnotes

1 http://www.ebi.ac.uk/ena
