# Supplementary material for: Influence of CO2 Degassing on the Microbial Community in a Dry Mofette Field in Hartoušov, Czech Republic (Western Eger Rift)
Source: Front Microbiol. 2018 Nov 21;9:2787. doi: 10.3389/fmicb.2018.02787 (PMC6258768; doi:10.3389/fmicb.2018.02787)
Supplement: Supplementary file 13 [file Data_Sheet_13.PDF]

SUPPLEMENTARY MATERIAL

Influence of CO_2 degassing on the microbial community in a dry mofette field in Hartoušov, Czech Republic (western Eger Rift)

Qi Liu[1], Horst Kämpf[2], Robert Bussert[3], Patryk Krauze[1], Fabian Horn[1], Tobias Nickschick[4], Birgit Plessen[5], Dirk Wagner[1, 6], Mashal Alawi[1]*

[1]GFZ German Research Centre for Geosciences, Helmholtz Centre Potsdam, Section 5.3 Geomicrobiology, Germany
[2]GFZ German Research Centre for Geosciences, Helmholtz Centre Potsdam, Section 3.2 Organic Geochemistry, Germany
[3]TU Berlin, Institute of Applied Geosciences, Germany
[4]University of Leipzig, Institute for Geophysics and Geology, Germany
[5]GFZ German Research Centre for Geosciences, Helmholtz Centre Potsdam, Section 5.2 Climate Dynamics and Landscape Evolution, Germany
[6]University of Potsdam, Institute of Earth and Environmental Sciences, Potsdam, Germany

Figures

Figure S1. Photo of a nearby wet mofette at the HMF. Bubbles indicate emerging CO_2.

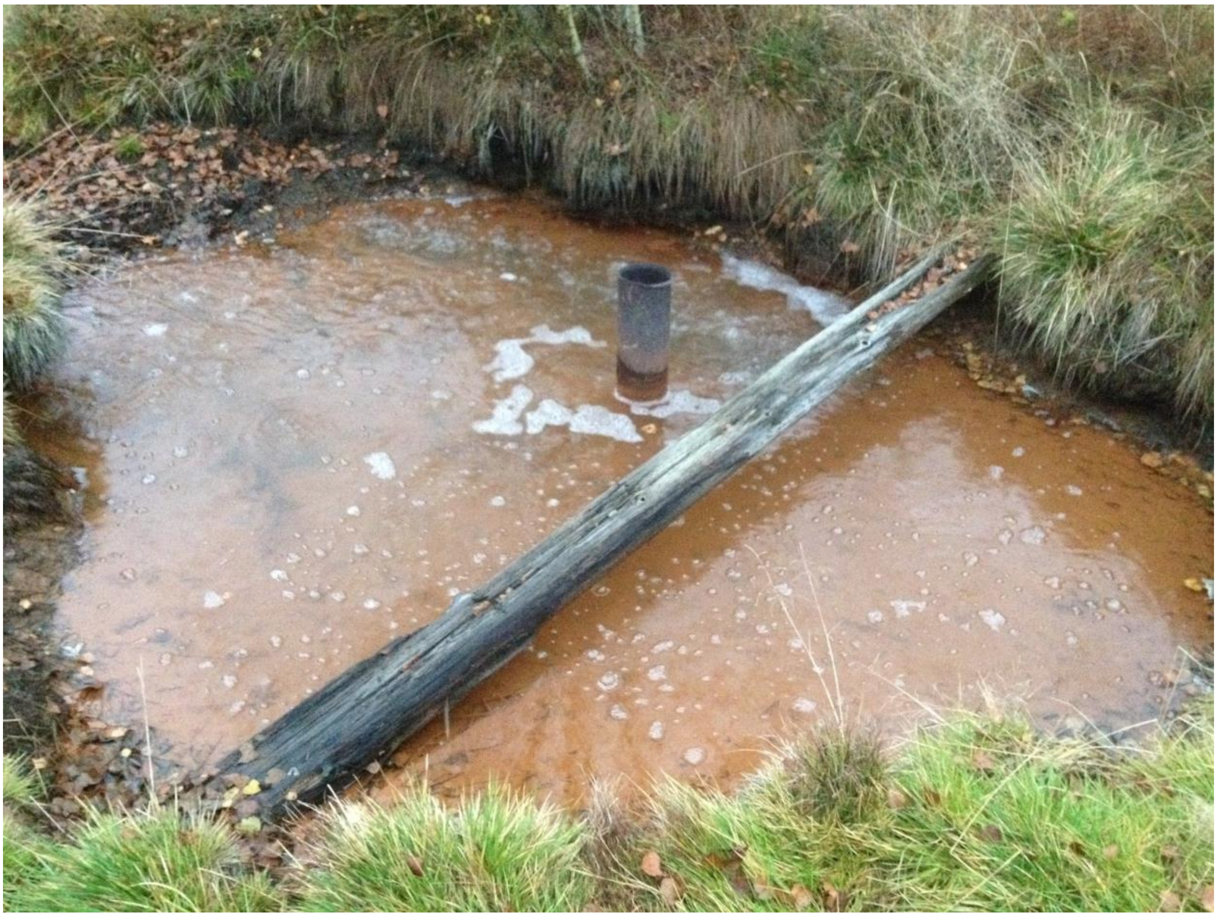

Tables S1-S12 (separate CSV files)

Table S1. Anion/cation concentrations, TOC, $\delta^{13}C_{org}$, pH, conductivity, water content, qPCR results, Shannon H index and Shannon Evenness index in the **mofette**.

Table S2. Anion/cation concentrations, TOC, $\delta^{13}C_{org}$, pH, conductivity, water content, qPCR results, Shannon H index and Shannon Evenness index at the **reference site**.

Table S3. Read numbers.

Table S4. Shannon H indices with different rarefaction thresholds.

Table S5. Matrix of the correlation results / **mofette**. The *p* values are in the upper-right and the R values are in the lower-left of the table.

Table S6. Matrix of the correlation results / **reference site**. The *p* values are in the upper-right and the R values are in the lower-left of the table.

Table S7. OTU distribution.

Table S8. ClusterA: Bray–Curtis dissimilarity values (depths between 0 – 95 cm).

Table S9. ClusterB: Bray–Curtis dissimilarity values (depths between 100 – 275 cm).

Table S10. Number of OTUs (relative abundances) occurring either only at the mofette or the reference site, or are present at both sites.

Table S11. The *Bonferroni* corrected p_{adj} values of the environmental parameters in CCA analyses.

Table S12. Comparison of bacterial communities at 10-20 cm between HMF and La Sima (Sáenz de Miera et al., 2014).
